# EZH2 Mutations Are Related to Low Blast Percentage in Bone Marrow and -7/del(7q) in De Novo Acute Myeloid Leukemia

**DOI:** 10.1371/journal.pone.0061341

**Published:** 2013-04-17

**Authors:** Xiuli Wang, Haiping Dai, Qian Wang, Qinrong Wang, Yang Xu, Ying Wang, Aining Sun, Jia Ruan, Suning Chen, Depei Wu

**Affiliations:** 1 Jiangsu Institute of Hematology, Key Laboratory of Thrombosis and Hemostasis of Ministry of Health, The First Affiliated Hospital of Soochow University, Suzhou, People's Republic of China; 2 Division of Hematology/Oncology, Department of Medicine, Weill Cornell Medical College, New York, New York, United States of America; University of North Carolina at Chapel Hill, United States of America

## Abstract

The purpose of the present work was to determine the incidence and clinical implications of somatic EZH2 mutations in 714 patients with de novo acute myelogenous leukemia by sequencing the entire coding region. EZH2 mutations were identified in 13/714 (1.8%) of AML patients were found to be more common in males (*P* = 0.033). The presence of EZH2 mutations was significantly associated with lower blast percentage (21–30%) in bone marrow (*P*<0.0001) and -7/del(7q) (*P* = 0.025). There were no differences in the incidence of mutation in 13 genes, ASXL1, CBL, c-KIT, DNMT3A, FLT3, IDH1, IDH2, MLL, NPM1, NRAS, RUNX1, TET2, and WT1, between patients with and without EZH2 mutations. No difference in complete remission, event-free survival, or overall survival was observed between patients with and without EZH2 mutation (*P*>0.05). Overall, these results showed EZH2 mutation in de novo acute myeloid leukemia as a recurrent genetic abnormality to be associated with lower blast percentage in BM and -7/del(7q).

## Introduction

Acute myeloid leukemia (AML) is a heterogeneous group of neoplastic disorders with considerable variability with regard to clinical course and response to treatment, as well as in their cytogenetic and molecular genetic features. Major advances in the understanding of the mechanisms for AML have been made by the characterization and the study of acquired cytogenetic abnormalities, especially reciprocal translocations and inversions observed in AML. Cytogenetic abnormalities are been considered the most important prognostic parameters in AML. Seven recurrent balanced chromosomal abnormalities, specifically t(15;17)(q22;q12), t(8;21)(q22;q22), inv(16)(p13.1q22) or t(16;16)(p13.1;q22), t(9;11)(p22;q23), t(6;9)(p23;q34), inv(3)(q21q26.2) or t(3;3)(q21;q26.2), and t(1;22)(p13;q13), are recognized as the cytogenetic hallmarks of genetically defined disease entities in the WHO classification criteria, which were revised in 2008. [1] Nevertheless, approximately half of patients with de novo AML lack typical clonal karyotypic abnormalities. [2]–[3] In recent years, genome-wide profiling of DNA copy-number variations and candidate gene sequencing have described abundant recurrent genetic mutations in patients with AML, such as FLT3, c-KIT, NPM1, WT1, ASXL1, DNMT3A, IDH1, IDH2, TET2, CBL, RUNX1, PHF6, and CEBPA. [4] Remarkably, several genes involved in the epigenetic regulation of transcription, including TET2, ASXL1, IDH1, IDH2, and DNMT3A, have been found to be mutated in AML, myelodisplastic syndrome (MDS) and other myeloproliferative neoplasmas (MPN). [5]–[12].

EZH2, located in 7q36.1, is another important gene associated with epigenetic regulation of transcription. EZH2 encodes the catalytic component of the polycomb repressive complex 2 (PRC2), which is responsible for the methylation of lysine 27 on the N-terminal tail of histone H3 (H3K27), which influences stem cell renewal by epigenetic modification. [13]–[14] Macro- and microdeletions involving EZH2 have been detected in about 10% of MDS patients [15], and a few MDS patients have shown loss-of-heterozygosity caused by acquired uniparental disomy. [16]–[17] Somatic mutations of EZH2 were recently identified in lymphoma and myeloid neoplasmas. In lymphoma, somatic mutations affecting codon 641 of EZH2 were detected in 7% of follicular lymphomas and 22% of diffuse large cell B-cell lymphomas of germinal center origin, resulting in enhanced lymphomagenesis. [18] In myeloid neoplasms, mutations were found throughout the EZH2 and have been described in 10–13% of poor-prognosis myelodysplasia-myeloproliferative neoplasms (MDS/MPN), 13% of myelofibrosis (MF), and 6% of MDS. [16]–[17] However, the prevalence and clinical significance of somatic EZH2 mutations in patients with acute myelogenous leukemia (AML) remains largely unknown.

In this study, we investigated the prevalence and prognostic value of somatic EZH2 mutations in 714 patients with de novo AML by PCR amplification of the entire coding region of EZH2 followed by direct bidirectional DNA sequencing. Patients were also assessed for the presence of mutations in 13 genes, including ASXL1, CBL, c-KIT, DNMT3A, FLT3, IDH1, IDH2, MLL, NPM1, NRAS, RUNX1, TET2, and WT1.

## Methods

### Patients

From January 2005 to December 2010, a total of 714 patients who had been newly diagnosed with AML at Jiangsu Institute of Hematology (JIH) were enrolled in the present study. Patients with antecedent hematologic diseases, especially agnogenic hematocytopenia, and those experiencing MDS, MDS/MPN, or therapy-related AML were excluded. In order to exclude AMLs attributable to MDS, all the samples from patients with karyotypes -7, 7q-, -5, 5q-, 20q-, or BM-blast less than 30% were examined retrospectively, and no evidence of morphologic myelodysplasia or atypical localization of immature progenitor (ALIP) was found. Diagnosis and classification of AML were defined according to the French-American-British classification (FAB) system and were revised using the World Health Organization (WHO 2008) classification system. The main characteristics of the patients in this study and the entire group are summarized in **Table S1**. This study was approved by the Ethics Committee of the First Affiliated Hospital of Soochow University. Written informed consent was provided for sample preservation and genetic analysis from every subject. For minors (56 patients, age 8–17 y) written informed consent of the parent or guardian was also obtained. Genomic DNA was extracted from frozen bone marrow mononuclear cells (BMMCs) after Ficoll gradient centrifugation using standard procedures.

For acute promyelocytic leukemia (APL) patients with t (15;17), all-trans-retinoic-acid- and arsenic-trioxide-based treatments were used in induction and consolidation therapy. Patients with non-APL AML were induced with standard first-line treatment, specifically the DA-like regimen, which consisted of daunorubicin (45 mg/m^2^, d1–3) and cytarabine (100–150 mg/m^2^, d1–7). With regard to the consolidation therapy, high-dose cytarabine based chemotherapy was performed on young patients. In addition, 27 patients received allogeneic hematopoietic stem cell transplantation (allo-HSCT) or autologous HSCT.

### Mutational Analysis of EZH2

EZH2 mutations were analyzed by PCR amplification of the entire coding region of 20 EZH2 exons followed by direct bidirectional DNA sequencing as previously described. [16]–[17] Patients were also assessed for the presence of mutations of ASXL1 [19], CBL [19], c-KIT [20], DNMT3A [21], FLT3 [19], IDH1 [19], IDH2 [19], MLL [22], NPM1 [19], NRAS [19], RUNX1 [19], TET2 [19], and WT1 [19] as previously reported. Abnormal sequencing results were confirmed by at least 2 repeated analyses.

### Statistical Analysis

Patient characteristics were analyzed by chi-square (*x* ^2^) or Fisher exact tests for univariate analysis. Kaplan-Meier analysis was used to evaluate patient survival. The log-rank test was used to compare survival difference. Binary logistic regression and COX model was used for the multivariate analysis of CR, EFS and OS, respectively. *P*-values less than 0.05 were deemed significant. All calculations were performed using the SPSS software package (version 13.0).

## Results and Discussion

### EZH2 Mutations in de novo AML

In this study, a total of 14 EZH2 mutations were documented in 13 patients with de novo AML. These involved exons 2, 9, 13, 17, and 18 (one case each) and exons 4, 12, 14, and 20 (2, 2, 3, and 2 cases, respectively). In this way, the frequency of somatic EZH2 mutations was 13/714 (1.8%) in de novo AML patients analyzed. The EZH2 mutations detected in the present study are listed in **Table 1** and depicted graphically in **Figure S1**. Nonsense, frameshift, and missense mutations accounted for 7.1% (1/14), 28.6% (4/14), and 64.3% (9/14) of all of EZH2 mutations, respectively, showing a heterozygous pattern. Mutation of EZH2 Y641 was not found in this group of patients. Four out of fourteen mutations were located in the conserved catalytic SET domain (amino acids 618–731), which is essential to the methyltransferase activity of EZH2. We analyzed the genomic DNA of 3 EZH2-mutated cases upon diagnosis and again during remission. Genomic DNA confirmed the somatic origin of EZH2 mutations (frame-shift mutations: Tyr297fs, Glu731fs, Cys534fs; missense mutation: His516Asn).

**Table 1:** Characteristics of 13 AML patients with EZH2 mutations.

No. cases	Sex	Age	BM blast (%)	Karyotype	Mutation(s)	Exon	Type of mutation	Protein level	Status	Other mutations
1	M	34	96.7	46, XY [20]	c.65_66delAG	2	frame-shift	p.Glu22fs	N	NPM1: A-type
										IDH2: c.419>A/p.Arg140Gln
										NRAS: c.G35>T/p.GLY12Val
2	M	50	96.2	46,XY, t(15;17)(q22;q12) [20]	c.278A>G	4	missense	p.Asn93Ser	N	
3	M	50	56	46, XY [20]	c.1978T>G	17	missense	p.Leu660Val	N	NPM1: B-type
										IDH2: c.419>A/p.Arg140Gln
										TET2: c.767C>G/p.A256G
4	M	41	51.5	46, XY [20]	c.278A>G	4	missense	p.Asn93Ser	N	ASXL1: c.2254dupG/p.Ala752fs
										IDH1: c.C394T/p.R132C
5	F	12	26.5	48,XX,-7,+8,t(11;12)(q12;p13),+mar1,+mar2 [20]	c.1615C>T	14	nonsense	p.Gln539X	N	
6	M	22	46	46, XY [20]	c.2003C>T	18	missense	p.Ala668Val	N	
7	M	37	91.5	46,XY,t(15;17)(q22;q12) [20]	c.2186_2187insGCCCCG	20	frame-shift	p.Ile730fs	N	
8	M	42	25.9	45,X,-Y,t(8;21)(q22;q22) [20]	c.891delT+insGGCATA	9	frame-shift	p.Tyr297fs;	N	C-KIT: c.85G>T/p.D816Y
					c.2190_2191dupATC	20	frame-shift	p.Glu731fs		MLL-PTD: mutated
9	M	36	89.5	47,XY,-8,+i(8q)*2 [20]	c.1600_1601dupT	14	missense	p.Cys534fs	N	FLT3-ITD: mutated
										RUNX1: c.415C>T/p.Arg140X
10	F	57	63	46, XY [20]	c.1445A>G	12	missense	p.Lys482Arg	N	
11	M	73	21	46, XY [20]	c.1484G>C	13	missense	p.Ser495Thr	N	
12	M	53	51.3	45,X,-Y,t(8;21)(q22;q22) [20]	c.1546C>A	14	missense	p.His516Asn	N	C-KIT: c.105T>G/p.N822K
										ASXL1: c.327G>T/p.E108D
13	M	39	20.8	46,xy,del(7q),der(8)(p?),der(7)i(7q-) [18]/48,idem,der(8)(p?),+19 [2]	c.1394C>A	12	missense	p.Ser465Tyr	N	

M, male; F, female; R, reported; N, novel.

### Correlation of EZH2 Mutations with Clinical and Cytogenetic Features

A comparison of clinical characteristics of patients with and without EZH2 mutations is shown in **Table 2**. Analysis of gender distribution in EZH2-mutated AML patients showed that EZH2 mutations in male patients (11/396; 2.8%) were 4.6 times more common (11/396; 2.8%) than in female patients (2/318; 0.6%) with AML (P = 0.033). Patients with EZH2 mutations showed lower BM blast counts (*P*<0.0001), than patients without EZH2 mutations. Among de novo AML patients with 20–30% of blast in BM, 14.3% (4/28) of them harbored EZH2 mutations. However, EZH2 mutations were detected in only 1.3% (9/686) of de novo AML patients with >30% of blast in BM. No difference in frequency of EZH2 mutations was observed between adult and pediatric patients (2.4% vs. 1.8%, *P*>0.05).

**Table 2:** Comparison of clinical and laboratory features between AML patients with and without EZH2 mutation.

Patient characteristics	Total no. cases (%)	EZH2 mutated No. (%)	EZH2 wild-type No. (%)	*P*
Median age, y (range)	43.0 (8.0–83.0)	41.0 (12.0–73.0)	43.0 (8.0–83.0)	0.754
WBC,×10^9^/L, median (range)	28.9 (0.66–490)	19.2 (1.4–167.4)	29.0 (0.7–490.0)	0.675
Hb, median (range)	83 (24–164)	73 (46–92)	83.0 (24.0–164.0)	0.189
Plt, ×10^9^/L, median(range)	35 (3.0–530)	38.0 (10.0–105.0)	35.0 (3.0–530)	0.887
BLAST%, median (range)	79.95 (11.5–99.4)	51.5 (18–96.7)	80.0 (11.5–99.4)	0.151
Gender				0.033
Male	396/714 (55.5)	11 (2.8)	385 (97.2)	
Female	318/714 (44.5)	2 (0.6)	316 (99.4)	
Age (y)				0.505
<16	41/714 (5.7)	1 (2.4)	40 (97.6)	
16–60	526/714 (73.7)	11 (2.1)	515 (97.9)	
>60	147/714 (20.6)	1 (0.7)	146 (99.3)	
WBC count, ×10^9^/L				0.946
<4.0	66/475 (13.9)	1 (1.5)	65 (98.5)	
4.0–30.0	177/475 (37.3)	3 (1.7)	174 (98.3)	
>30.0	232/475 (48.8)	3 (1.3)	229 (98.7)	
Hb, g/L				0.165
<20	57/456 (12.5)	2 (3.5)	55 (96.5)	
20–100	272/456 (59.6)	5 (1.8)	267 (98.2)	
>100	127/456 (27.9)	0/7 (0)	127 (100.0)	
PLT count, ×10^9^/L				0.743
<20.0	122/454 (26.9)	1 (0.8)	121 (99.2)	
20.0–100.0	269/454 (59.3)	5 (1.9)	264 (98.1)	
>100.0	63/454 (13.9)	1 (1.6)	62 (98.4)	
BM-blast (%)				<0.0001
<30%	28/714 (3.9)	4 (14.3)	24 (85.7)	
≥30%	686/714 (96.1)	9 (1.3)	677 (98.7)	
Karyotype				0.988
normal	360/698 (51.6)	6(1.7)	354 (98.3)	
t(8;21)	59/698 (8.5)	2(3.4)	57 (96.6)	
t(15;17)/t (11;17)	93/698 (13.3)	2(2.2)	91 (97.8)	
inv(16), t(16;16)	24/698 (3.4)	0(0)	24 (100.0)	
tri8	11/698 (1.6)	0(0)	11 (100.0)	
complex abnormalities	74/698 (10.6)	2(2.7)	72 (97.3)	
hyperdiploid	8/698 (1.1)	0(0)	8 (100.0)	
t(9;22)	17/698 (2.4)	0(0)	17 (100.0)	
11q23/MLL	10/698 (1.4)	0(0)	10 (100.0)	
others	38/698 (5.4)	1(2.6)	37 (97.4)	
Risk statusψ				0.887
Better-risk	176/698 (25.2)	4(2.3)	172 (97.7)	
Intermediate-risk	417/698 (59.7)	7(1.7)	410 (98.3)	
Poor- risk	105/698 (15.0)	2(1.9)	103 (98.1)	
-7,7q-				0.025
with	26/698(3.7)	2(7.7)	24 (92.3)	
without	672/698(96.3)	11(1.6)	661 (98.4)	

ψ Risk status: Better-risk: inv(16)/t(16;16), t(8;21),t(15;17); Intermediate-risk: normal, +8, t(9;11), other undefined risk; Poor-risk: complex, −5, 5q−, −7, 7q−, 11q23(non t(9;11)), inv(3), t(3;3), t(6;9), t(9;22).

(A) Structure of the EZH2 protein and location of EZH2 mutations. (B) DNA sequencing chromatograms of AML genomic DNA samples showing 14 mutations in 13 AML patients.

Among 714 AML patients undergoing cytogenetic analysis, karyotypic data were available in 698 cases. The remainder (n = 16) of cases lacked karyotypic data due to lack of metaphase. Out of 698 patients supplied with cytogenetic data, abnormal karyotypes were detected in 338 cases (47.9%). EZH2 status was correlated to cytogenetics as summarized in **Table 2**. EZH2 mutations were revealed in patients with normal karyotype in 6 cases, -7/del(7q) accompanied by complex karyotypes in 2 cases, t(15;17)(q22;q12) in 2 cases, t(8;21)(q22;q22) in 2 cases, and i(8q) in 1 case. There was no difference in the incidence of EZH2 mutations among patients with favorable karyotype (4/176, 2.3%), intermediate-risk karyotype (7/417, 1.7%), or unfavorable karyotype (2/105, 1.9%; *P* = 0.887). Of note, EZH2 mutations were observed much more frequently in the cases with -7/del(7q) (2/26, 7.7%) than in the cases without -7/del(7q) (11/672, 1.6%; *P* = 0.025). No relationship was observed between EZH2 mutations with other cytogenetic abnormalities.

### Association of EZH2 Mutations with Other Molecular Abnormalities

To investigate the interactions of gene mutations in the leukemogenesis of de novo AML, a comprehensive mutational screening of EZH2 and 13 other genes was performed. After excluding known polymorphisms and silent mutations, FLT3-ITD mutations were found in 25.0%, NPM1 in 22.5%, TET2 in 10.2%, IDH2 in 9.7%, N-RAS in 8.2%, FLT3-TKD in 6.5%, C-KIT in 6.1%, DNMT3A in 5.3%, WT1 in 4.6%, ASXL1 in 4.5%, IDH1 in 4.2%, MLL-PTD in 3.3%, RUNX1 in 2.5%, and CBL in 0.8% of all cases examined. No association was found between EZH2 mutations and other molecular abnormalities in the present study.

### Impact of EZH2 Mutations on Response to Therapy and Clinical Outcome

**Figure 1:**
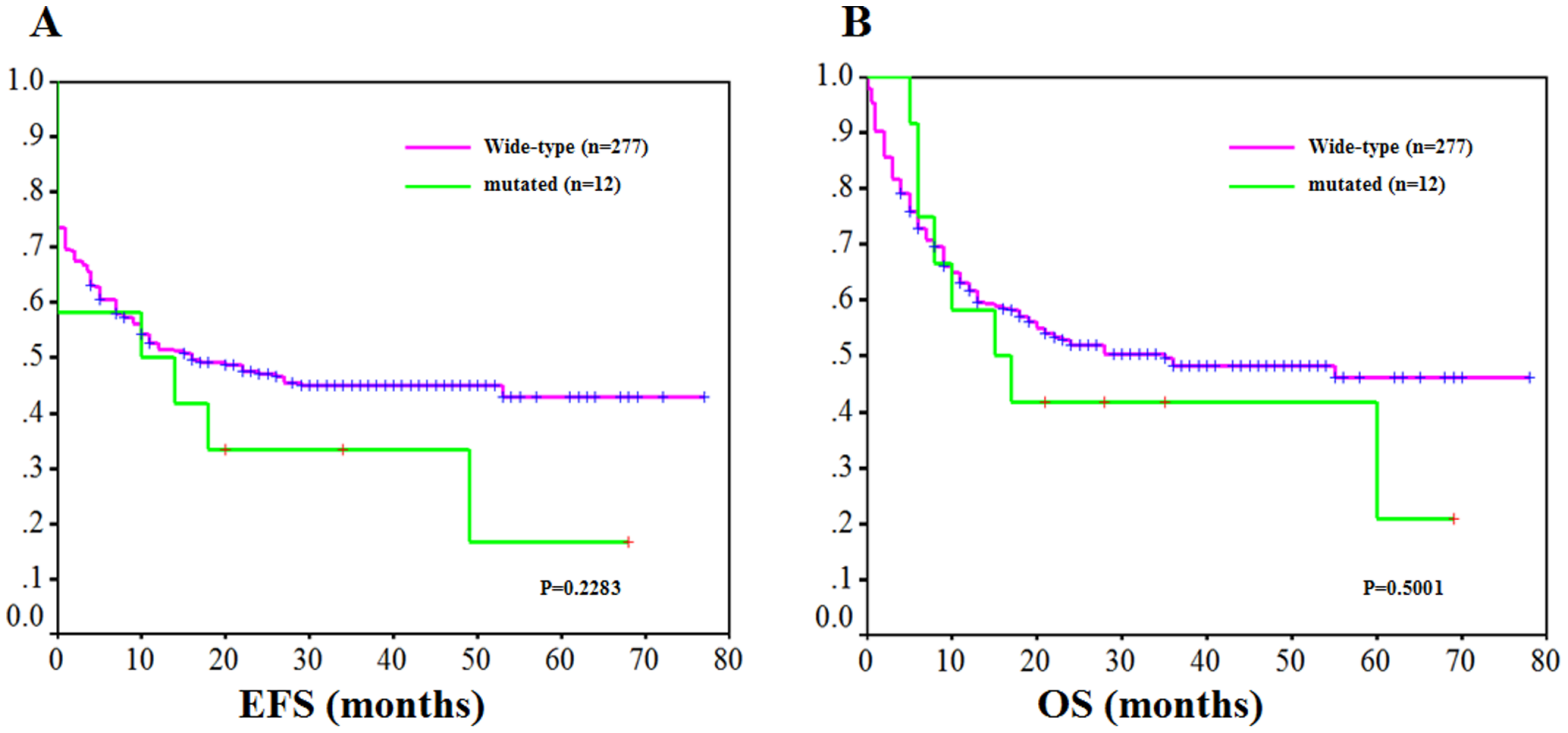
Kaplan-Meier survival curves according to EZH2 mutation status. (A) EFS in de novo AML patients according to EZH2 mutations. The green line represents patients with mutated EZH2 (n = 12); and magenta line, patients with unmutated EZH2 (n = 277; *P* = 0.2283); (B) OS in de novo AML patients according to EZH2 mutations. The green line represents patients with mutated EZH2 (n = 12); and the magenta line represents patients with unmutated EZH2 (n = 277; P = 0.5001).

For correlation with clinical outcome, patients with APL were excluded. EZH2 mutations had no influence on achieving complete remission (CR) (**Figure 1**). The CR rates of cases with and without EZH2 mutations were not significantly different (61.5% vs. 75.0%, *P* = 0.329). No significant difference in event-free survival (EFS) (3 year rates, 33.1% vs. 45.2%; *P* = 0.2283) or overall survival (OS) (3 year rates, 41.0% vs. 49.1%; *P* = 0.5001) with a median follow-up of 46.6 months (range, 1–79 months) were observed.

Multivariate analysis showed FLT3-ITD, NRAS, and risk-status are independent influence factors with regard to CR rate (*P*<0.001, p = 0.004, *P* = 0.001 respectively). In a multivariate Cox regression model, mutations of FLT3-ITD, age, risk-status, and N-RAS (only associated with OS) showed independent prognostic significance (EFS: *P* = 0.002, = 0.002, <0.001; OS: *P* = 0.001, = 0.005, <0.001, = 0.023).

Collectively, the results of the present study demonstrate that EZH2 mutations could be detected in a substantial proportion of patients with de novo AML and that these mutations occurred more frequently in male patients than in female patients. No difference in CR rates, EFS, or OS was observed between AML patients with and without EZH2 mutation. The presence of EZH2 mutations in AML was found to be closely associated with lower BM blast percentage (20–30%) and -7/del(7q). In the FAB classification of MDS, patients with 21–30% of blasts in BM were defined as having refractory anemia with excess blasts in transformation (RAEB-t). [23] The WHO classification criteria lowered the threshold to 20% for the number of blasts required for the diagnosis of AML. [1] This arbitrary threshold in blast percentage eliminated RAEB-t in MDS and patients in this category were considered to have AML. However, several reports have argued that the biology of RAEB-t is distinct from that of AML and should be considered a subtype of MDS. [24]–[25] In the present study, results showed the frequency of EZH2 mutations in patients with 21–30% of BM blasts to be much higher than that in patients with >30% of blasts in BM (14.3% versus 1.3%, *P*<0.0001). In addition, EZH2 is located on the distal part of chromosome 7q. EZH2 mutations were observed more frequently in cases with -7/del(7q) than in cases without -7/del(7q) (7.7% versus 1.6%, *P* = 0.025). It was concluded that somatic mutations of EZH2 might play an important role in pathogenesis of de novo AML patients with -7/del(7q) or with 21–30% of blasts in BM, which is classified as RAEB-t in FAB classification. Because of rarity of EZH2 mutations in de novo AML, the prognostic impact of EZH2 mutations in AML is still uncertain, and will need to be assessed in larger cohorts of patients collected on multi-center co-operative studies, though there no significant difference in EFS or OS was observed between EZH2 mutated patients and wild-type in the present study.

## Supporting Information

Figure S1 **EZH2 mutations in AML patients.**
(TIF)

Table S1 **Clinical characteristics of 714 patients with de novo AML.**
(DOC)
