# Supplementary figures and images for: EZH2 Mutations Are Related to Low Blast Percentage in Bone Marrow and -7/del(7q) in De Novo Acute Myeloid Leukemia
Source: PLoS One. 2013 Apr 17;8(4):e61341. doi: 10.1371/journal.pone.0061341 (PMC3629223; doi:10.1371/journal.pone.0061341)

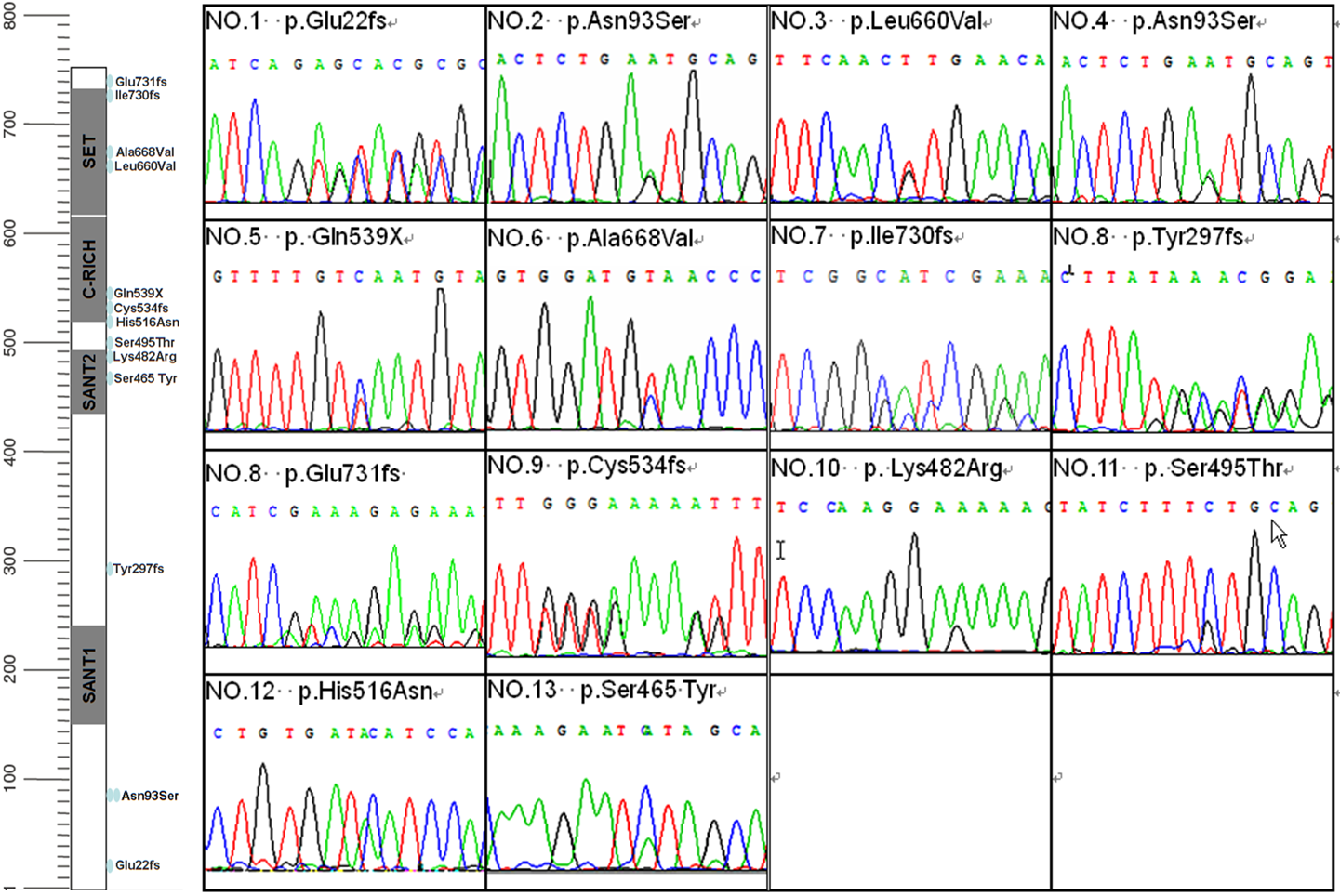

Supplement: Figure S1 — EZH2 mutations in AML patients. (TIF) [file pone.0061341.s001.tif]
