# Supplementary material for: EZH2 Mutations Are Related to Low Blast Percentage in Bone Marrow and -7/del(7q) in De Novo Acute Myeloid Leukemia
Source: PLoS One. 2013 Apr 17;8(4):e61341. doi: 10.1371/journal.pone.0061341 (PMC3629223; doi:10.1371/journal.pone.0061341)
Supplement: Table S1 — Clinical characteristics of 714 patients with de novo AML. (DOC) [file pone.0061341.s002.doc]

Supplemental Table 1 Clinical characteristics of 714 patients with de novo AML.

| Characteristics | Value (median, range) |
| --- | --- |
| Number of patients, no. | 714 |
| median age, y(range) | 43.0(8.0-83.0) |
| sex ratio (M/F) | 1.2(396:318) |
| Clinical features |  |
| Peripheral blood |  |
| WBC,×10^9^/L, median(range) | 28.9(0.66~490) |
| Hb, median(range) | 83(24~164) |
| Plt, ×10^9^/L, median(range) | 35(3.0~530) |
| BM-Blast%, median(range) | 79.95(11.5~99.4) |
| FAB subtype |  |
| M0, no. (%) | 9/714(1.2) |
| M1, no. (%) | 119/714(16.7) |
| M2, no. (%) | 217/714(30.4) |
| M3, no. (%) | 127/714(17.8) |
| M4, no. (%) | 95/714(13.3) |
| M5, no. (%) | 119/714(16.7) |
| M6, no. (%) | 22/714(3.1) |
| M7, no. (%) | 0/714(0) |
| Unclassified, no. (%) | 6/714(0.9) |
| Cytogenetics |  |
| Normal karyotype, no. (%) | 365/714(51.1) |
| Abnormal karyotype, no. (%) | 333/714(46.6) |
| Unsuccessful karyotype, no. (%) | 16/714(2.2) |

WBC, white blood cell; Hb, hemoglobin; PLT, platelet; BM, bone marrow.
